# Clinical Outcomes and Complication Rates of Endodontically Treated Teeth with Fixed Dental Prostheses: A Retrospective Study

**DOI:** 10.3390/dj13010042

**Published:** 2025-01-20

**Authors:** Sarah Aloqayli, Hanin Alsalhi, Ali Alenezi

**Affiliations:** 1 College of Dentistry, Qassim University, Buraydah 51452, Saudi Arabia; 361203666@qu.edu.sa; 2 Department of Conservative Dental Sciences, College of Dentistry, Qassim University, Buraydah 51452, Saudi Arabia; han.alsalhi@qu.edu.sa; 3 Department of Prosthetic Dental Sciences, College of Dentistry, Qassim University, Buraydah 51452, Saudi Arabia

**Keywords:** complications, crown, endodontics, nonvital, survival rate

## Abstract

**Background/Objectives**: Endodontically treated teeth (ETT) often have significant structural damage and require multiple reinforcing methods during the reconstruction process. The aim of this study was to evaluate the complication rates of ETT with and without a post. **Methods**: The study investigated various clinical factors, including technical complications as well as biological complications. All patients who had previously received fixed dental prostheses (FDPs) were subjected to clinical and radiographical examinations during their follow-up visits. Clinical and radiographic assessments were performed to determine the cumulative survival rate, and life table survival analyses of FDPs in the presence of complications were performed. **Results**: A total of 287 ETT were evaluated in this study, in which 219 were placed in females and 68 in males, with an average follow-up time of 82.4 months. There were no significant differences between ETT with a post and ETT without a post regarding the complication rates. The complication rates of the FDPs without a post revealed a survival rate of 58% after 5 years, 44% after 10 years, and 29% after 15 years. On the other hand, the complication rates of the FDPs with a post revealed survival rates of 50%, 30%, and 21% after 5, 10, and 15 years, respectively. The Kaplan–Meier survival analysis showed no significant differences in the complication rates, regardless of the existing of the posts (*p* = 0.830). **Conclusions**: ETT restored with FDPs with or without a post can show similar complication rates in a long-term evaluation. Furthermore, the types of final restoration may not have an effect on the complication rate with ETT.

## 1. Introduction

Endodontic therapy is commonly recommended when teeth have been subjected to various forms of damage, including decay, trauma, and periapical lesions [1]. Its main objective is to extend the lifespan of a functional tooth by addressing or preventing apical periodontitis. Its success, however, is influenced by several factors, such as the soundness of the tooth’s structure during root canal preparation and the quality of the tooth after its eventual restoration [2,3]. Ensuring the success of root canal treatment necessitates that an endodontically treated tooth be assessed both clinically and radiographically. This treatment’s success and the functionality of the treated tooth should be confirmed by scheduling patients for regular follow-up appointments. The failure of endodontic treatment is influenced by many factors, among which the common ones are the persistence of bacteria, inadequate root canal filling, the overextension of root-filling materials, improper coronal sealing, and missed canals [4].

Previous studies indicated success rates greater than 90% for nonsurgical root canal therapy under controlled conditions [5,6]. The reduced fracture resistance of teeth that have undergone root canal treatment is potentially caused by alterations in the mechanical attributes of dentin, fluctuations in moisture content, the passage of time, and a decrease in proprioception, among various other factors [7]. Typically, teeth subjected to endodontic treatment often have significant structural damage and therefore require reinforcement via multiple methods during reconstruction. A common approach involves using a post to help support the core restoration [8]. Prefabricated posts are frequently employed because of their affordability and ease of use. A custom-fabricated post and core can also be utilized.

Although prefabricated posts are a common choice given their convenience, many clinicians prefer fabricating custom cast posts and cores as a method of restoring extensively damaged teeth [9,10,11]. Commonly used posts can be categorized on the basis of material composition: metal, fiber, and ceramic posts. Metal posts can be further classified as conventional cast posts or prefabricated metal posts. Choosing an appropriate post system can be challenging, owing to various factors that need to be considered, such as a tooth’s position in the arch, the amount of remaining dental structure, the presence of contact points, and the type of restoration to be installed [12,13]. Ideally, a post and a core should aid coronal retention while being safe, biocompatible, and having a high tensile strength, as well as fatigue resistance against occlusal and shear loads [14,15,16].

Several studies have suggested that post placement may not be necessary for all ETT, particularly cases with minimal tooth loss. Recent developments in adhesive dentistry can also be adopted to enhance the retention of restorations after root canal treatment. On this basis, the necessity of a post depends on factors such as the amount of remaining coronal structure, functional requirements, and the type of tooth being treated. In situations wherein there is insufficient coronal tooth structure and ferrule preparation is infeasible, a post is often recommended to prevent further compromising the tooth’s structure [4].

Before fitting a permanent prosthetic restoration onto a root-filled tooth, the preprosthetic treatment of the remaining tooth structure is frequently necessary. This requirement typically stems from significant defects in the coronal hard tissue of root-filled teeth even before treatment, which must be addressed before the final restoration can be installed [17,18]. The importance of crown coverage in the long-term survival of ETT has been a topic of debate. Some authors have suggested that the long-term survival rate increases when a crown is placed [19]. In selecting the best possible restoration for a root-filled tooth, an essential task is to consider various interconnected factors, among which some of the crucial determinants include the prevention of microbial leakage into a canal, the restoration of form and function, occlusal stability, the protection of the residual tooth structure, and adequate esthetics [20,21].

In consideration of the above-mentioned challenges, this retrospective clinical study was conducted to evaluate the technical and biological complication rates of ETT restored with fixed dental prostheses or direct restorations. Restorations with and without posts and cores were compared over an extended follow-up period. The null hypotheses were that no differences would be found in the complication rates between ETT restored with and without posts.

## 2. Materials and Methods

This study was conducted at the dental clinics of Qassim University’s College of Dentistry and was granted approval by the university’s Ethics Committee in 2021 (Approval code: ST/6094/17-4-2021). This study was conducted in accordance with the Preferred Reporting Items for Observational studies in Endodontics (PROBE) 2023 guidelines (Table S1) [22]. Radiographic and clinical examinations were performed for patients visiting the dental clinics from January to August 2023. All patients who met all the inclusion criteria and signed the consent form were included in this study. The inclusion criteria were patients above 18 years of age who were able to provide informed consent and patients with one or more endodontically treated teeth which were restored with single crowns, conventional bridges, or direct restorations. Patients with vital teeth, removable prostheses, cantilever bridges, or implant-supported fixed dental prostheses (FDPs) were excluded.

The researchers independently performed both clinical and radiographic assessments to identify complications related to the patients’ teeth. Periapical radiographs of all restorations were obtained with the standardized long-cone technique. Clinically, the restorations were evaluated via visual inspection. In addition, the handle of the mirror was used to assess the mobility of the tooth by applying pressure to the tooth from the buccal and lingual aspects and also vertically down the long axis of the tooth. ETT with fixed prosthetics or direct restorations were grouped under the “survival” group if no clinical or radiographic signs of complications were present. Teeth were classified as having complications when they exhibited associated objective issues, such as fractures, secondary caries, post/prosthetic de-bonding, or radiologic signs of failure indicating root or post fracture, such as radiolucent lines inside the root canal or the post, atypical gaps between the canal wall and the intracanal material, an enlargement of the periodontal ligament space along the peri-radicular surfaces, angular bone loss, step-like bone defects, V-shaped diffuse bony lesions, or root resorptions aligned with the fracture line prior to the distinct separation of the fractured fragment. Following the examinations, the researchers collected data on the types of treatment, the types of post and core, the condition of ETT, and complication categories.

The study investigated various clinical factors, including technical complications (e.g., fractures and de-bonding) and biological complications (e.g., caries and periapical lesions). The data were categorized on the basis of gender, the location of ETT (maxilla or mandible), the area where ETT were found (anterior, anterior–posterior, and posterior), the presence of a post, the type of post (fiber, metal, or casted), the type of final restoration (single crowns, fixed bridges or connected crowns, and direct restorations), and the type of material (metal-ceramic (MC) or all ceramic). All types of all-ceramic FDPs were analyzed as one collective group, and so were all post systems based on fiber materials. The same approach was applied to different posts fabricated using metals.

### Statistical Analyses

The data were statistically examined using the Statistical Package for the Social Sciences version 28 (SPSS Inc., Chicago, IL, USA), and complication-free survival was analyzed using the Kaplan–Meier method. Specifically, the ETT survival rate was investigated on the basis of the cumulative incidence of technical and biological complications. Life table survival analyses were conducted to evaluate the cumulative survival rate of ETT under the presence of complications. Significant differences between the evaluated groups were analyzed using the Mann–Whitney U test, with *p* ≤ 0.05 considered as statistically significant with 95% confidence intervals (CIs).

## 3. Results

**Figure 1:**
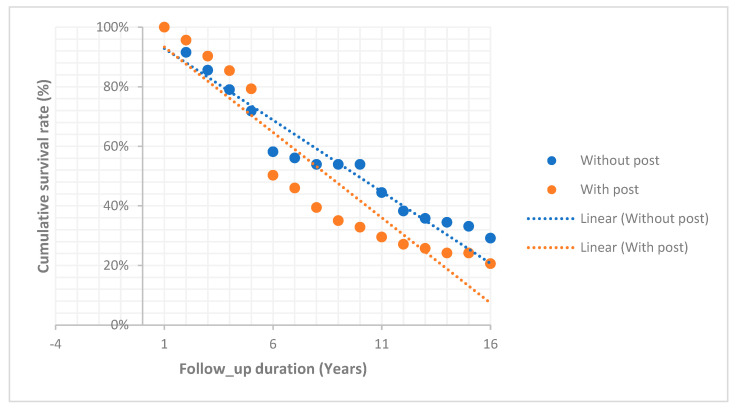
Kaplan–Meier survival function of endodontically treated teeth with or without post restorations for the incidence of complications, (*p*-value = 0.830).

Table 1 summarizes the findings of the examinations, which covered 287 ETT. Of these treatments, 219 involved females and 68 involved males. Patients’ ages ranged from 18 to 84 years old. The average follow-up period was 82.4 months. A notable disparity in ETT between the genders was observed. The endodontic treatment of the females resulted in a relatively higher complication rate (63.5%) than that of the males (58.8%). Nevertheless, the difference was not statistically significant (*p* = 0.490). In Figure 1, the Kaplan–Meier survival analysis revealed no significant differences among endodontically treated teeth with or without post restorations for the incidence of complications, (*p*-value = 0.830).

With regard to the type of final restoration, the majority of the treated teeth (64.5%) were restored with single crowns. Fixed bridges or connected crowns accounted for 23% of the cases, while direct restorations made up 12.5%. These variations in distribution were not statistically significant (*p* = 0.102). The fact that ETT were located along the jaw had no significant effect on the rate of complication (*p* = 0.182), and the areas of ETT (anterior, anterior–posterior, posterior, or cross-arch) had no statistically significant differences (*p* = 0.333). Approximately half of the ETT (50.9%) had no posts, whereas the remaining proportion (49.1%) were fitted with such implements. No statistically significant difference in complication rates was found between these groups (*p* = 0.797).

The evaluation of post type distribution showed that metal posts were the most prevalent materials (70.9%), followed by fiber posts (17.7%), and cast posts (11.3%). No statistically significant variations in distribution were found (*p* = 0.319). The majority of the examined FDPs were constructed using the MC material, accounting for 70.5% of the total. These MC-FDPs exhibited a slightly increased rate of complications compared with that observed in full ceramic prostheses. However, this disparity did not reach a statistically significant level (*p* = 0.115).

Table 2 and Table 3 show the results of the life table survival analyses, specifically the cumulative survival rates of ETT with or without post restorations over time. Without posts, ETT exhibited a survival rate of 58% after 5 years, 44% after 10 years, and 29% after 15 years. ETT with posts showed cumulative survival rates of 50%, 30%, and 21% after 5, 10, and 15 years, respectively. Table 4 presents the complications (technical and biological) observed in the evaluated groups. ETT in male patients had a higher rate of technical complications (13.23%) than ETT in their female counterparts (6.85%). However, the difference between the two groups was not statistically significant (*p* = 0.0729). Female patients suffered a higher rate of biological complications (56.62%) than the males (45.58%), but no statistically significant difference was found (*p* = 0.0830). As for complication rates based on the patient’s age, significantly fewer mechanical complications were seen in patients who were aged 31 to 40 years old compared to patients from other age groups (*p* = 0.021).

**Figure 2:**
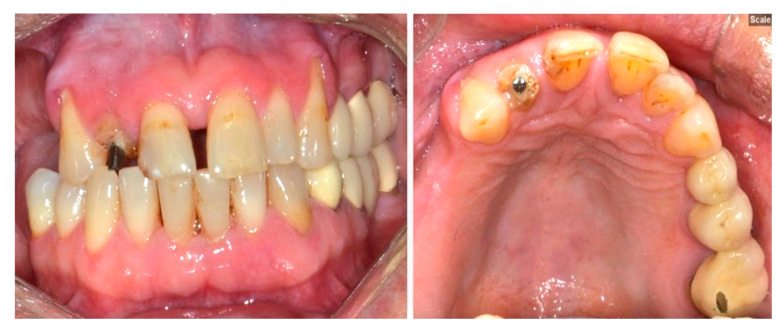
Clinical images showing fractured dental restorative material with the loss of material on the endodontically treated tooth 12. Ceramic chippings can be seen on the occlusal surface on the MC bridge from 24–26.

With respect to the final treatment, patients who were fitted with bridges or connected crowns experienced more technical complications (13.63%) than those who had direct restorations (8.33%) or single crowns (6.48%). However, the difference was not statistically significant (*p* = 0.267). The highest rate of biological complications was found in bridges or connected crowns (57.57%), followed by single crowns (55.13%), and direct restorations (41.66%). Even so, no statistically significant difference was found among these treatment groups (*p* = 0.197). Figure 2 indicates that some clinical images show fractured dental restorative material, with the loss of material on the endodontically treated later incisor (tooth #12).

In relation to the type of materials constituting fixed prostheses, those made with MC showed a higher rate of mechanical complications (9.03%) than all-ceramic prostheses (4.05%). However, the difference in complication rates was not statistically significant (*p* = 0.219). Conversely, all-ceramic prostheses suffered from a higher rate of biological complications (62.16%) than those made with MC (45.76%), but again, the difference was not statistically significant (*p* = 0.197). The analysis of ETT location indicated that teeth located at the maxilla had a higher rate of mechanical complications (9.03%) than those located on the mandible (4.05%). This difference was statistically significant (*p* = 0.004). In regard to biological complications, ETT on the mandible exhibited a statistically significantly higher rate than ETT at the maxilla (*p* = 0.007828).

In terms of prosthesis site, posterior prostheses suffered from mechanical complications the most (10.88%), followed by anterior–posterior prostheses (6.25%), and anterior prostheses (1.61%). The difference in mechanical complications was statistically significant (*p* = 0.004). Anterior prostheses exhibited the highest rate of biological complications (67.74%), followed by anterior–posterior prostheses (51.29%), and posterior prostheses (43.75%). The difference was also statistically significant (*p* < 0.001). ETT with posts exhibited a higher rate of mechanical complications (9.22%) than those without posts (7.53%). However, the difference was not statistically significant (*p* = 0.533). In contrast, prostheses without posts had a slightly higher rate of biological complications (54.10%) than prostheses with posts (53.90%). Nevertheless, no statistically significant difference was found between these groups (*p* = 0.381).

Fiber posts showed the greatest incidence of mechanical complications (40%), followed by metal and cast posts (13% and 6.25%, respectively). However, this difference was not statistically significant (*p* = 0.095). Metal posts had the highest biological complication rate (72%), followed by cast and fiber posts (62.5% and 52%, respectively). Similarly, no statistically significant difference was found among these rates (*p* = 0.056).

## 4. Discussion

As previously stated, this research assessed the rates of complication in ETT with fixed dental prostheses and compared them with ETT fitted with and without posts and cores. The data were obtained using a retrospective study design, which may show risks of obtaining inconsistent data, but this was not a problem in this research because the evaluation and data collection for all patients included in this study were recorded at the same dental clinics in 2023.

The study investigated 287 cases of ETT with final restorations (146 cases of teeth restored with posts and 141 cases restored without posts). The number of cases was selected randomly during a predetermined period and is similar to those in other reports [9,22,23]. Some clinical studies examined the different types of posts over the mid- to long-term horizon with a small sample of patients [9,23,24], whereas Balkenhol et al. [25] reported numerous cases of ETT, with a total of 802 cast posts and cores analyzed. The evaluation method based on clinical and radiographical examinations enabled the current work to detect the complications associated with FDPs. The size of the final sample was considered suitable for drawing general conclusions regarding the association of these complications with the types of treatment that the patients received.

This research inquired into the long-term biological and technical complications of ETT. Posts and cores are commonly used to restore severely damaged teeth, which may affect the long-term prognosis of these teeth. In relation to this, the mean survival rates presented in this study may be acceptable because technical and biological complications are mostly treatable and may not lead to treatment failure. The evaluation period of around 80 months was longer than that in other investigations [24,25], leading to the assumption that the findings are representative. Nevertheless, employing a longer evaluation period would be more practical and allow for the better understanding of biological and technical complications associated with ETT.

The findings indicated 10-year cumulative survival rates of around 44% for ETT with posts and 30% for ETT without posts. A comparison of studies may be difficult given the variances in methodologies, populations, materials, and follow-up periods. According to the literature, different factors affect the prognosis of ETT restored with posts. These factors include the material, length, and diameter of a post as well as the core material used [26,27]. In addition, Wegner et al. reported in their clinical study that remaining coronal walls can play a crucial role in the performance of posts and fracture resistance [28]. A long-term clinical evaluation by Raedel et al. showed a 10-year survival rate of 75.7% for teeth treated with cast posts and cores [29]. Many researchers have recommended that post placement be considered mainly for ETT with limited remaining coronal walls [30,31,32].

Among the patients in the current work, the majority of cases were found in female patients, accounting for 76.3% (219 ETT) of the total, whereas the cases in males represented 23.7% (68 ETT). Despite the unequal distribution of genders, the analysis showed no significant differences in the results with respect to this factor. This contrasts with the literature, wherein considerable variations in complication rates based on gender are reported. For instance, a retrospective study conducted by Gómez-Polo et al. uncovered a higher failure rate of ETT in women compared with men after a follow-up of more than eight years [9]. This contradicts the findings of another clinical study on 638 patients treated with 788 posts, wherein a significantly higher failure rate was found among men [33].

The current findings showed that prosthesis location had no significant impact on survival rate. This is in agreement with a recent study by Frankenberger and colleagues [34]. The fact that the majority of the examined cases were on the maxilla (58.9%) corresponds with the results of other studies [9,35], although the distribution of the examined cases on different teeth should be expected when a high number of cases is evaluated. With regard to prosthesis site, most of the treated teeth were located in the posterior region. The fixed prostheses on posterior teeth showed a slightly lower complication rate (62.2%) than that observed in the prostheses on anterior teeth (69.4%) after more than 80 months of follow-up. However, no significant difference was detected between these sites (*p* = 0.333). Some studies have also suggested that maxillary premolars are associated with a higher risk of failure compared with other sites [9,21]. In the current research, prostheses on teeth along the maxilla showed a higher incidence of mechanical complications, with twenty out of one hundred and sixty nine cases (11.83%) suffering from these problems, but those on teeth located along the mandible showed an incidence of four out of one hundred and eighteen cases (3.38%).

Teeth restored with single crowns exhibited lower complication rates than teeth restored with bridges after 80 months of follow-up. The Kaplan–Meier analysis revealed survival rates of less than 50% after 10 years for all the fixed prostheses examined. These rates are lower than that reported by Selby et al., who found a survival rate of 85.1% after 10 years of function [36]. Note, however, that survival was defined in the present study as the absence of complications upon examination, including minor and treatable complications. Some reports have indicated a strong relationship between the survival of ETT and complete crown placement, probably because of the shielding effect of crowns [37]. ETT without complete crown coverage may be at a risk of failure that is six times greater than that projected for EET with complete crowns [38].

Teeth restored with fixed bridges exhibited the highest complication rate (71.2%) after long-term evaluation. The multi-unit FDPs in function are believed to be associated with a high risk of loss retention of a restoration [39]. This risk can be attributed to the difficulty in alignment among multiple tooth preparations, which may cause excessive tapering that complicates retention [36]. Some reports suggested that the bending moments of prosthetic systems are one of the main contributors to the higher rate of failure of FDPs compared with single crowns [25].

The present research also revealed that biological complications with fixed bridges accounted for a higher percentage, with thirty-eight out of sixty-six cases (57.57%) exhibiting these issues compared with only nine out of sixty-six cases (13.63%) having mechanical complications. The latter were restored with direct fillings, and they also suffered the least complications among fixed prostheses, although the difference was not statistically significant (*p* = 0.197384). Similar clinical success rates after a three-year follow-up period were found in a prospective clinical investigation of ETT restored with direct composite restorations and full-coverage crowns [40].

The distribution of cases on the basis of post presence was approximately equal between the two groups, with 146 cases (50.9%) having no posts and 141 cases (49.1%) restored with posts. Out of 141 cases with posts, 13 (9.22%) had mechanical complications, which were higher than that observed among cases without posts (i.e., 11 out of 146 (7.53%)). This difference can be attributed to the fact that cases requiring posts typically have more extensive tooth damage [41,42]. Interestingly, a study carried out by Willershausen et al. suggested that the prognosis of ETT can be improved by avoiding the insertion of metal posts [43]. In their retrospective, non-randomized cohort study, the authors evaluated 775 ETT and found a 115% higher risk of complications following post insertion compared with teeth restored without posts [43].

In the present study, the statistical analysis revealed no significant difference in survival rates between post materials after more than 75 months of follow-up. These findings correspond with the results of several clinical investigations [9,23]. A 10-year retrospective study reported similar survival rates between cobalt chrome cast posts and prefabricated titanium posts after a mean 10-year follow-up period [9]. In the current study, around 80% of the fixed prostheses with posts (regardless of type) were free of complications after five years of evaluation. This percentage dropped to around 40% after 10 years, which was less than the percentage found in fixed prostheses without posts (around 50%). A long-term controlled clinical study was conducted by Fokkinga et al. to evaluate 17-year survival data regarding different metal post and core restorations with covering crowns [44]. They found that the 17-year survival rates of these restorations vary from 71% to 92%. Nevertheless, they concluded that the type of core restorations under the covering crowns of ETT has no influence on survival probabilities [44].

The distribution of cases in the present study indicated that complications were associated most commonly with metal posts, followed by fiber posts. However, Gbadebo et al. reported success rates of 97.5% and 100% for metallic stainless steel posts and glass fiber posts, respectively, in a six-month follow-up period [45]. These findings suggest that glass fiber posts outperform metallic posts in the short term. Conversely, Marielle Dias Martins et al. [46] found that the failure rates of fiber and metal posts are similar when they are used in ETT restoration. Additionally, a randomized controlled trial conducted by Rafael Sarkis-Onofre et al. [47] revealed that glass fiber and cast metal posts exhibited good and comparable clinical performance, even after a follow-up period of up to nine years. Currently, conventional cast posts and cores are being replaced by newer post and core materials. Nowadays, commonly used materials are prefabricated posts made of fiber-reinforced resin composite and adhesively retained metal. Figueiredo et al. [48] conducted a systematic review and meta-analysis to delve into the incidence rates of root fractures in different post-retained restorations reported in clinical trials and cohort studies. The results of the meta-analysis revealed a survival rate of 90% for metal-based posts and 83.9% for fiber-reinforced posts, albeit no significant differences were found between these implements with regard to root fracture incidence [48]. In a randomized controlled trial, Ferrari et al. [49] found that customized fiber posts may have lower success rates than those offered by solid fiber posts after six years of follow-up. To date, clinical data are inconclusive as to which post material ensures the best prognosis for post-retained restorations [21]. Posts are used mainly for teeth with severe loss of coronal tooth walls to improve coronal retention. Additional controlled clinical studies are needed to ascertain which particular post materials or post designs can offer the highest long-term survival rates.

In the present research, a larger proportion of mechanical complications was associated with MC prostheses, specifically 16 out of 177 cases (9.03%). By contrast, only three out of seventy-four all-ceramic prostheses (4.05%) had such complications. A recent systematic review evaluated the survival rates of multi-unit prostheses fabricated with MC and all-ceramic materials [50]. The review uncovered a better survival rate for MC prostheses than that found for all-ceramic prostheses five years after insertion. In this study, biological complications, such as caries and periapical lesions, were the most commonly found complications (in 54% of the cases) after around 80 months of follow-up. Mechanical complications, such as fracture or de-ponding, were found in 8.4% of the cases. Note, however, that many of these technical and biological complications can be regarded as minor failures because they are solvable in most of the cases [51]. The literature showed substantial variations with regard to the most frequently reported complications in ETT restored with posts and FDPs. In a systematic review, the majority of failure events were caused by post de-bonding and a loss of retention in single crowns [37]. A prospective randomized controlled trial reported many cases of root fractures (with metal posts) and post–core–crown complex loosening (with fiber-reinforced restorations) after five years of service [2]. Conversely, the occurrence of biological complications in this study was more prevalent in all-ceramic prostheses, accounting for 46 out of 74 cases (62.16%), in comparison with PFM prostheses, which accounted for 81 out of 177 cases (45.76%). Complications such as root fracture, the loss of restorative seal, the dislodgement of the core, and periodontal injury due to biological width invasion during margin preparation are commonly associated with structurally compromised teeth [52]. A tooth’s structural integrity is compromised when an access cavity is prepared for endodontic treatment. This can lead to increased cusp deflection during tooth function and, consequently, a greater likelihood of fractures [53]. Moreover, when endodontic treatment is combined with the loss of tooth structure, i.e., mesio–occluso–distal (MOD) cavities, the situation may be aggravated due to the significant loss of structure [54,55]. Meanwhile, several studies suggested that the amount of remaining tooth structure can be a crucial factor for the survival of restorations following endodontic treatment [19,55]. Mergulhão and colleagues [56] discovered that among endodontically treated teeth with restored MOD cavities, the most common type of failure is unrepairable, such as axial fractures. In contrast, for intact teeth, the primary mode of failure is repairable.

One of the limitations of this study is the retrospective character of the evaluation process, which indicates that no detailed history of the complications could be guaranteed. For example, the presence of apical periodontitis before root canal treatment was not identified and excluded from biological complications. Furthermore, the findings of this study were based on clinical and radiographical evaluations of previous treatments that were performed by different clinicians at different time periods.

The estimated survival rates were based on the absence of complications and not failed treatment. Some of the limitations of this study are that it did not investigate the influence of remaining coronal tooth structure on the rate of complications. Thus, all the findings should be interpreted with respect to the limitations of this study. The evaluation of survival rates in a controlled clinical trial with a prospective study design and a large study population may reveal higher rates. It would be interesting, from a clinical perspective, to consider whether a post is essential for ETT. The present study found no difference in survival rates between fixed prostheses with and without posts, implying that post insertion does not need to be recommended regularly for ETT. These findings may help in the decision-making process of a suitable treatment plan after root canal treatment.

## 5. Conclusions

ETT with and without posts can suffer from similar complications in the long term, and the types of final restoration (direct or indirect) may have no effect on complication rates. A higher risk of technical complications can be found in fixed prostheses placed in the upper arch compared with those installed in the lower arch. The risk of biological complications can be higher in the lower arch than in the upper arch. More controlled long-term clinical studies are needed to confirm the findings of the present research.

## Figures and Tables

**Table 1:** Descriptive data of the endodontically treated teeth included in this study, with follow-up time between the different factors. The statistical unit is the complication rate.

Groups		Number of Endodontically Treated Teeth (%)	Mean Observation Period (Months) ± SD	Cases with Complication (%)	*p*-Value
Patient’s gender	Female	219 (76.3%)	82.8 ± 71.7	139 (63.5%)	0.49
	Male	68 (23.7%)	78.8 ± 65.5	40 (58.8%)	
Age group	18–30	42 (13.8%)	83.52 ± 33.24	30 (71.4%)	0.083
	31–40	63 (20.7%)	82.56 ± 73.56	28 (44.4%)	
	41–50	91 (29.9%)	76.08 ± 58.68	59 (64.8%)	
	51–60	71 (23.4%)	105.96 ± 91.56	41 (57.7%)	
	More than 60	20 (6.6%)	104.4 ± 60.24	16 (80%)	
Type of final treatment	Single crown	185 (64.5%)	82.9 ± 71.8	114 (61.6%)	0.102
	Bridge or connected crowns	66 (23%)	83.2 ± 71.7	47 (71.2%)	
	Direct restoration	36 (12.5%)	80.4 ± 62.4	18 (50%)	
Type of Materials for Prosthesis	MC	177 (70.5%)	83.6 ± 72.1	97 (54.8%)	0.115
	All Ceramic	74 (29.5%)	82.4 ± 71.5	49 (66.2%)	
Location	Maxilla	169 (58.9%)	82.4 ± 71.5	100 (59.2%)	0.182
	Mandible	118 (41.1%)	82.1 ± 71.2	79 (66.9%)	
Site	Anterior	62 (21.6%)	81.6 ± 67.9	43 (69.4%)	0.333
	Posterior	193 (67.2%)	82.7 ± 71.5	120 (62.2%)	
	Anterior/posterior	32 (11.2%)	83.2 ± 71.7	16 (50%)	
Presence of Post	Without Post	146 (50.9%)	82.4 ± 71.5	90 (61.6%)	0.797
	With post	141 (49.1%)	76.6 ± 60.9	89 (63.1%)	
Type of Post	Fiber	25 (17.7%)	81.3 ± 66.4	13 (52%)	0.319
	Metal	100 (70.9%)	76.8 ± 61.1	67 (67%)	
	Cast	16 (11.4%)	76.8 ± 61.03	9 (56%)	

**Table 2:** Life table survival analysis showing the cumulative survival rate for the complications associated with endodontically treated teeth with post restorations.

Interval Start Time (Year)	Number Entering Interval (ETT)	Number Withdrawing During Interval (ETT)	Number Exposed to Risk (ETT)	Number of Terminal Events (ETT)	Proportion Terminating (%)	Proportion Surviving (%)	Cumulative Proportion Surviving at End of Interval (%)	Std. Error (%)
0	146	0	146	0	0	100	100	0
1	146	8	142	12	8	92	92	2
2	126	8	122	8	7	93	86	3
3	110	10	105	8	8	92	79	4
4	92	8	88	8	9	91	72	4
5	76	5	74	14	19	81	58	5
6	57	2	56	2	4	96	56	5
7	53	1	53	2	4	96	54	5
8	50	3	49	0	0	100	54	5
9	47	0	47	0	0	100	54	5
10	47	3	46	8	18	82	44	5
11	36	0	36	5	14	86	38	5
12	31	1	31	2	7	93	36	5
13	28	1	28	1	4	96	34	5
14	26	0	26	1	4	96	33	5
15	25	0	25	3	12	88	29	5

**Table 3:** Life table survival analysis showing the cumulative survival rate for the complications with endodontically treated teeth without post restorations.

Interval Start Time (Year)	Number Entering Interval (ETT)	Number Withdrawing during Interval (ETT)	Number Exposed to Risk (ETT)	Number of Terminal Events (ETT)	Proportion Terminating (%)	Proportion Surviving (%)	Cumulative Proportion Surviving at End of Interval (%)	Std. Error (%)
0	141	0	141	0	0	100	100	0
1	141	7	138	6	4	96	96	2
2	128	6	125	7	6	94	90	3
3	115	7	112	6	5	95	85	3
4	102	9	98	7	7	93	79	4
5	86	8	82	30	37	63	50	5
6	48	2	47	4	9	91	46	5
7	42	0	42	6	14	86	39	5
8	36	0	36	4	11	89	35	5
9	32	0	32	2	6	94	33	5
10	30	1	30	3	10	90	30	5
11	26	3	25	2	8	92	27	5
12	21	3	20	1	5	95	26	5
13	17	1	17	1	6	94	24	5
14	15	0	15	0	0	100	24	5
15	15	3	14	2	15	85	21	4

**Table 4:** Types of complications that were found on the evaluated groups.

Groups		Endodontically Treated teeth with Technical Complications (%)	*p*-Value	Endodontically Treated teeth with Biological Complications (%)	*p*-Value
Patient’s gender	Female	15/219 (6.9%)	0.072	124/219 (56.6%)	0.0831
	Male	9/68 (13.2%)		31/68 (45.6%)	
Age group	18–30	8/42 (19%)	0.021	22/42 (52.4%)	0.63
	31–40	1/63 (1.6%)		27/63 (42.9%)	
	41–50	6/91 (6.6%)		59/91 (64.9%)	
	51–60	5/71 (7%)		36/71 (50.8%)	
	More than 60	3/20 (15%)		13/20 (65%)	
Type of final treatment	Single crown	12/185 (6.5%)	0.267	102/185 (55.1%)	0.197
	Bridge or connected crowns	9/66 (13.6%)		38/66 (57.6%)	
	Direct restoration	3/36 (8.3%)		15/36 (41.6%)	
Type of Materials for The Prosthesis	MC	16/177 (9%)	0.219	81/177 (45.8%)	0.201
	All Ceramic	3/74 (4.1%)		46/74 (62.2%)	
Location	Maxilla	20/169 (11.8%)	0.004	100/169 (59.2%)	0.007
	Mandible	4/118 (3.4%)		75/118 (63.6%)	
Site	Anterior	1/62 (1.6%)	<0.001	42/62 (67.7%)	<0.001
	Posterior	21/193 (10.9%)		99/193 (51.3%)	
	Anterior/posterior	2/32 (6.3%)		14/32 (43.6%)	
Presence of Post	Without Post	11/146 (7.5%)	0.533	79/146 (54.1%)	0.381
	With post	13/141 (9.2%)		76/141 (53.9%)	
Type of Post	Fiber	10/25 (40%)	0.095	13/25 (52%)	0.056
	Metal	13/100 (13%)		72/100 (72%)	
	Cast	1/16 (6.3%)		10/16 (62.5%)	
Total		24/287 (8.4%)		155/287 (54%)	

## Data Availability

Data collected and interpreted in this study are maintained by the authors and can be made available upon request.

## Supplementary Materials

The following supporting information can be downloaded at the following website: https://www.mdpi.com/article/10.3390/dj13010042/s1, Table S1: Checklist of items to be included when reporting observational studies according to Preferred Reporting Items for Observational studies in Endodontics (PROBE) 2023 guidelines.
